# Haematolohical Profile of Subacute Oral Toxicity of Molybdenum and Ameliorative Efficacy of Copper Salt in Goats

**DOI:** 10.4103/0971-6580.72676

**Published:** 2010

**Authors:** Kusum, R. Raina, P. K. Verma, N. K. Pankaj, V. Kant, J. Kumar, A. K. Srivastava

**Affiliations:** Divisions of Pharmacology and Toxicology, Faculty of Veterinary Sciences and Animal Husbandry, SKUAST, R.S. Pura, Jammu, Jammu and Kashmir, India; 1 Divisions of Veterinary Physiology, Faculty of Veterinary Sciences and Animal Husbandry, SKUAST, R.S. Pura, Jammu, Jammu and Kashmir, India

**Keywords:** Ammonium molybdate, copper sulfate, goats, hematological profile

## Abstract

Molybdenum toxicity produces a state of secondary hypocuprosis, resulting into alterations in normal hematological profile. In the present study, ammonium molybdate alone and with copper sulfate (II) pentahydrate (ameliorative agent) was administered orally for 30 consecutive days in healthy goats of group 1 and 2, respectively, to access the effect on the hematological profile on different predetermined days of dosing. Administration of ammonium molybdate alone produced significant decline in the mean values of hemoglobin (Hb), packed cell volume (PCV), total leukocyte count (TLC), total erythrocyte count (TEC), and mean corpuscular hemoglobin concentration (MCHC), with a significant increase in neutrophil level and mean corpuscular volume (MCV). However, values of erythrocyte sedimentation rate, mean corpuscular hemoglobin, and differential leukocyte count were not significantly altered. On comparing observations of ameliorative group with the group 1 goats, it is concluded that the ameliorative copper salt has beneficial effects in alleviating the alterations in the values of Hb, PCV, TLC, TEC, MCV, MCHC, and neutrophils.

## INTRODUCTION

Molybdenum (Mo) is an essential trace element. It is a component of xanthine oxidase, aldehyde oxidase, and sulfite oxidase.[1,2] Mo is required in amino acid and protein metabolism,[3] sulfur metabolism,[2] hydrolysis of phosphate esters, and transport and utilization of iron.[4] Forages grown in Mo-rich soil absorb and accumulate Mo more than their normal requirement[5] and animals consuming such forages develop molybdenosis. Intake of high levels of Mo in animal body produces a state of conditioned copper (Cu) deficiency, which in turn is responsible for functional integrity of hematopoietic system.[6,7] Therefore, Mo toxicity alters normal hematological profile, which can be rectified and/or prevented by using Cu salt as ameliorative agent.[3] Hypocuprosis secondary to Mo toxicity has been reported in cattle, sheep, rabbits, rats, guinea pigs, and poultry,[8–12] but the goat has not received adequate attention in this respect. The potential ameliorative effect of Cu supplementation is of preventive importance in Mo poisoning. Therefore, the present study has been designed to investigate in detail the alteration in hematological parameters during subacute oral toxicity of Mo.

## MATERIALS AND METHODS

A total of eight healthy female crossbred goats weighing 25 to 40 kg of 2 to 2.5 years of age were procured from local farmers of R.S. Pura, Jammu. They were acclimatized for two weeks in the college animal farm shed under hygienic conditions before the commencement of the experiment. The animals were maintained on *ad lib* water and green fodder of the season. The experimental protocol was approved by institutional ethics committee. The experimental goats were divided into two Groups of four each. Goats of Group 1 were used to study the changes in hematological parameters during subacute oral toxicity of Mo, in which ammonium molybdate, AR ((NH_4_)_6_Mo_7_O_24_.4H_2_O, Qualigenes Fine Chem. Ltd. Mumbai) alone was administered orally at the rate of 20 mg/kg/day (equivalent to 10.86 mg of Mo) for 30 consecutive days, and goats of Group 2 were used to study the efficacy of copper sulfate (II) pentahydrate on the changes produced in hematological parameters by subacute oral toxicity of Mo, in which same dose of ammonium molybdate along with copper sulfate (II) pentahydrate, AR (CuSo_4_.5 H_2_O, Hi-Media Laboratories Ltd.) at the rate of 7.9 mg CuSo_4_/Kg/day (equivalent to 2 mg of Cu) was administered orally for 30 consecutive days. Copper sulfate (II) pentahydrate was provided 30 to 40 minutes before ammonium molybdate administration. The daily administration of salts was made between 9.30 and 10.00 AM after dissolving them in adequate amount of tap water, based on the body weights of goats. The animals were weighed weekly and dosage of salts was corrected for changes in body weight. All animals were closely observed for clinical signs and mortality.

Blood samples (2 ml) were collected in clean sterile glass culture tubes containing dipotassium salt of EDTA (Hi-Media, Mumbai) at the rate of 2 mg/ml of blood on days 0, 1, 3, 7, 14, 21, 28, 30 of treatment and on days 7 and 14 after termination of treatment by jugular vein puncture for hematological analysis. Hemoglobin (Hb) was determined by Sahli’s method, packed cell volume (PCV) by microhematocrit method, erythrocyte sedimentation rate (ESR) by Wintrobe method, and total erythrocyte count (TEC), total leukocyte count (TLC), differential leukocyte count (DLC), mean corpuscular volume (MCV), mean corpuscular hemoglobin (MCH), and mean corpuscular hemoglobin concentration (MCHC) were determined by using standard reference methods of Benjamin.[13]

Hematological parameters on different days of administration of ammonium molybdate were compared with pre-exposure (0 day) value of the respective Group, and a probability level of *P*<0.05 was considered statistically significant.[14]

## RESULTS AND DISCUSSION

Repeated administration of ammonium molybdate alone for 30 consecutive days in Group 1 produced mild toxicosis characterized by inappetence, weight loss, decreased ruminal motility, rough hair coat, intermittent diarrhea, alopecia, sway back, anemia, achromotrichia, and emaciation. These clinical manifestations varied in severity among different animals of the Group. The toxic symptoms were not manifested in ameliorative (Group 2) animals. The details of the hematological profile of Group 1 and Group 2 are presented in Tables 1 and 2, respectively. Repeated oral administration of ammonium molybdate alone produced significant decline (*P*<0.01) in the mean value of Hb (gm/dl) and (*P*<0.05) PCV (%) on 30^th^ day (7.53 ± 0.23 and 24.75 ± 1.38, respectively) during dosing as compared with the pre-exposure values. The mean values of TLC (× 10^3^/mm^3^) and TEC (× 10^6^/mm^3^) also manifested significant decrease (*P*<0.05) from 28^th^ day onwards (7.35 ± 0.50 and 8.10 ± 0.43, respectively). The mean value of MCHC (%) manifested a significant decline (*P*<0.05) on day 7 onwards (26.37 ± 1.48) after termination of Mo administration as compared with 0-day level (36.12 ± 2.67). However, there was a significant rise in MCV (fl) and neutrophils level. The mean values of ESR (mm/24 hours), MCH (pg), lymphocytes, monocytes, eosinophils, and basophils (%) manifested no significant deviation during and after Mo administration.

**Table 1:** Effect of repeated oral administration of ammonium molybdate in group 1 goats for 30 consecutive days on hematological parameters (mean ± SE) (n = 4)

Parameter	Days									
	Treatment days								Post-treatment days	
	0	1	3	7	14	21	28	30	7	14
Hb (gm/dl)	9.75 ± 0.68	9.60 ± 0.89	9.65 ± 0.79	9.68 ± 0.69	9.88 ± 0.66	9.60 ± 0.25	7.85^a^ ± 0.26	7.53^b^ ± 0.23	7.08^b^ ± 0.19	7.40^b^ ± 0.21
PCV (%)	27.25 ± 1.93	27.75 ± 1.93	28.25 ± 0.85	28.00 ± 1.87	28.50 ± 1.94	27.50 ± 2.47	26.50 ± 3.07	24.75^a^ ± 1.38	27.00 ± 1.08	29.50 ± 1.44
TLC (× 10^3^/mm^3^)	9.00 ± 0.32	9.08 ± 0.33	9.15 ± 0.25	8.87 ± 0.20	8.88 ± 0.13	8.74 ± 0.33	7.35^a^ ± 0.50	6.87^a^ ± 0.31	7.07^b^ ± 0.22	7.01^b^ ± 0.32
TEC (× 10^6^/mm^3^)	10.57 ± 0.63	10.63 ± 0.46	10.11 ± 0.47	10.70 ± 0.46	10.99 ± 0.68	9.78 ± 0.77	8.10^a^ ± 0.43	7.85^a^ ± 0.23	7.96^b^ ± 0.32	8.26^a^ ± 0.39
Lymphocytes	56.26 ± 1.31	56.75 ± 0.75	54.50 ± 1.04	53.50 ± 1.19	52.75 ± 2.06	51.50 ± 1.71	47.75 ± 3.17	47.25 ± 1.65	53.50 ± 1.55	53.00 ± 2.86
Neutrophils	31.75 ± 0.85	31.50 ± 0.65	31.00 ± 0.91	32.00 ± 1.08	31.50 ± 1.32	32.50 ± 1.19	35.25 ± 1.49	35.00^a^ ± 0.91	37.25 ± 2.90	38.75 ± 3.57
Monocytes	4.50 ± 1.04	3.50 ± 0.87	3.25 ± 0.48	3.00 ± 0.71	3.50 ± 0.29	3.75 ± 0.48	3.50 ± 0.87	3.00 ± 0.58	2.75 ± 0.48	3.00 ± 0.71
Eosinophils	2.50 ± 0.29	1.25 ± 0.48	1.75 ± 0.63	2.00 ± 0.41	2.25 ± 0.63	2.75 ± 0.85	3.50 ± 0.65	3.25 ± 1.11	2.00 ± 0.91	3.25 ± 0.95
Basophils	1.25 ± 0.48	1.50 ± 0.29	1.50 ± 0.29	1.50 ± 0.29	1.00 ± 0.41	1.75 ± 0.48	1.75 ± 0.25	1.25 ± 0.25	1.75 ± 0.48	1.00 ± 0.41
MCV (fl)	25.83 ± 1.45	26.23 ± 2.11	28.23 ± 2.09	26.34 ± 2.13	26.29 ± 2.72	28.13 ± 1.08	33.38 ± 5.33	31.55 ± 1.68	34.22^a^ ± 2.60	36.12^a^ ± 3.35
MCH (pg)	9.25 ± 0.50	8.99 ± 0.49	9.59 ± 0.79	9.06 ± 0.54	9.07 ± 0.71	10.04 ± 0.97	9.82 ± 0.80	9.62 ± 0.46	8.92 ± 0.32	8.99 ± 0.30
MCHC (%)	36.12 ± 2.67	34.72 ± 2.52	34.12 ± 2.41	34.64 ± 1.41	35.55 ± 4.78	35.87 ± 3.60	30.87 ± 3.80	30.61 ± 1.48	26.37^a^ ± 1.48	25.31^a^ ± 1.60
ESR (mm/24 hours)	9.50 ± 0.65	8.25 ± 0.75	7.50 ± 0.65	7.25 ± 0.48	9.00 ± 0.41	9.25 ± 0.75	9.75 ± 0.48	10.50 ± 0.29	8.50 ± 0.96	9.00 ± 0.41

a,b Significantly different as compared with pre-exposure (0 day) value of the same group at 5% (*P*<0.05) and 1% (*P*<0.01), respectively; Hb – hemoglobin; PCV - packed cell volume; TLC - total leukocyte count; TEC - total erythrocyte count; MCV - mean corpuscular volume; MCH - mean corpuscular hemoglobin; MCHC - mean corpuscular hemoglobin concentration; ESR - erythrocyte sedimentation rate

**Table 2:** Effect of repeated oral administration of ammonium molybdate along with copper sulfate (II) pentahydrate in group 2 goats for 30 consecutive days on hematological parameters (mean ± SE) (n = 4)

Parameter	Days									
	Treatment days								Post-treatment days	
	0	1	3	7	14	21	28	30	7	14
Hb (gm/dl)	10.65 ± 0.33	10.78 ± 0.12	10.85 ± 0.17	10.73 ± 0.70	10.68 ± 0.31	10.45 ± 0.37	10.30 ± 0.45	10.35 ± 0.54	10.10 ± 0.52	10.63 ± 0.44
PCV (%)	28.75 ± 1.49	29.75 ± 1.11	29.50 ± 0.65	29.00 ± 1.58	30.75 ± 1.11	29.50 ± 0.65	28.00 ± 1.41	28.50 ± 1.56	29.00 ± 2.97	29.75 ± 2.10
TLC (× 103/mm3)	9.51 ± 0.35	8.54 ± 0.21	9.65 ± 0.39	9.73 ± 0.54	9.82 ± 0.60	9.80 ± 0.47	9.50 ± 0.58	8.51 ± 0.43	9.69 ± 0.48	9.77 ± 0.37
TEC (× 106/mm3)	11.02 ± 0.52	10.84 ± 0.62	10.91 ± 0.63	10.82 ± 0.52	11.06 ± 0.53	10.74 ± 0.67	10.52 ± 0.48	10.23 ± 0.56	10.30 ± 0.40	10.61 ± 0.25
Lymphocytes	57.50 ± 3.80	56.50 ± 1.19	60.75 ± 0.85	58.75 ± 1.89	57.75 ± 2.02	55.25 ± 1.11	49.75 ± 0.85	48.75 ± 1.38	55.00 ± 1.78	54.25 ± 2.06
Neutrophils	32.00 ± 1.29	35.00 ± 2.65	31.25 ± 1.70	32.75 ± 2.25	29.50 ± 2.02	28.75 ± 0.95	34.00 ± 3.34	35.50 ± 2.84	35.00 ± 1.96	33.50 ± 2.60
Monocytes	4.50 ± 0.65	4.75 ± 0.48	5.00 ± 0.71	4.75 ± 0.48	4.25 ± 0.48	4.25 ± 1.11	3.25 ± 0.48	3.00 ± 0.41	2.75 ± 0.75	4.50 ± 0.65
Eosinophils	2.25 ± 0.63	1.50 ± 0.29	1.75 ± 0.25	1.75 ± 0.48	1.75 ± 0.25	2.00 ± 0.91	2.75 ± 0.85	2.75 ± 0.48	2.50 ± 0.87	2.50 ± 0.65
Basophils	1.50 ± 0.29	1.25 ± 0.48	1.75 ± 0.25	1.75 ± 0.48	2.25 ± 0.25	2.00 ± 0.91	2.00 ± 0.41	1.75 ± 0.48	1.50 ± 0.29	1.25 ± 0.48
MCV (fl)	26.32 ± 2.13	27.69 ± 1.84	27.25 ± 1.34	27.15 ± 2.59	27.89 ± 0.88	27.78 ± 1.83	26.92 ± 2.37	27.93 ± 1.04	28.42 ± 3.39	28.07 ± 1.99
MCH (pg)	9.75 ± 0.63	10.02 ± 0.50	10.04 ± 0.60	9.91 ± 0.46	9.72 ± 0.53	9.89 ± 0.92	9.87 ± 0.68	10.25 ± 0.94	9.82 ± 0.42	10.05 ± 0.58
MCHC (%)	37.47 ± 2.93	36.36 ± 1.32	36.85 ± 1.15	37.54 ± 3.87	34.91 ± 2.00	35.43 ± 1.07	36.93 ± 1.54	36.67 ± 2.85	36.13 ± 4.65	36.41 ± 3.46
ESR (mm/24 hours)	8.50 ± 0.65	7.75 ± 0.63	7.75 ± 0.63	7.00 ± 0.71	8.00 ± 0.41	7.75 ± 0.85	7.50 ± 0.96	8.25 ± 0.63	8.25 ± 1.49	8.75 ± 0.95

a,b Significantly different as compared with pre-exposure (0 day) value of the same group at 5% (*P*<0.05) and 1% (*P*<0.01), respectively; Hb - hemoglobin; PCV - packed cell volume; TLC - total leukocyte count; TEC - total erythrocyte count; MCV - mean corpuscular volume; MCH - mean corpuscular hemoglobin; MCHC - mean corpuscular hemoglobin concentration; ESR - erythrocyte

In ameliorative Group (Group 2), repeated administration of ammonium molybdate along with Cu salt resulted in nonsignificant alterations in the values of mean plasma Hb, PCV, ESR, TLC, TEC, MCV, MCH, MCHC, and DLC during and after the experimental period of investigation.

The fall in Hb content in Group 1 goats can be attributed to the secondary Cu deficiency, which leads to impaired iron metabolism.[15] Similar findings have been reported from rats, guinea pigs, goats, buffalo calves, and cow calves consequent to Mo intoxication.[16–19] The transient decrease in PCV value might be attributed to the defective hematopoiesis and reutilization of endogenous iron as a result of Mo-induced Cu deficiency.[20] *Similar decrease in PCV was also observed by Mills et al*.[21] and Shakeel[22] in cow calves and in poultry birds, respectively. The decrease in TEC and TLC might have been due to the failure of hematopoiesis and defective iron metabolism.[23] Similar findings of low TEC have also been recorded in goats, cross-bred cow calves, and poultry.[19,22,24] Low leukocyte count has been reported in dairy cattle, cross-bred cow calves, and poultry.[19,22,25]

The increased MCV and decreased MCHC in subacute oral Mo toxicity in goats in the present study suggested macrocytic hypochromic anemia. Sharma[19] also reported elevation in the MCV, fall in MCHC, and no alteration in MCH in molybdenotic cross-bred cow calves. Similar observations of nonsignificant deviation in different leukocytes during and after Mo administration, except neutrophils, have been recorded by Sharma[17] in molybdenotic goats.

On comparing the observations of ameliorative Group with that of Group 1 goats, it is concluded that supplementation of ameliorative Cu salt along with Mo reversed most of the altered hematological parameters in molybdenotic goats.
